# Ultrasonic Propagation in Highly Attenuating Insulation Materials

**DOI:** 10.3390/s20082285

**Published:** 2020-04-17

**Authors:** David A. Hutchins, Richard L. Watson, Lee A.J. Davis, Lolu Akanji, Duncan R. Billson, Pietro Burrascano, Stefano Laureti, Marco Ricci

**Affiliations:** 1 School of Engineering, University of Warwick, Coventry CV4 7AL, UK; D.A.Hutchins@warwick.ac.uk (D.A.H.); r.watson.2@warwick.ac.uk (R.L.W.); lee.davis@warwick.ac.uk (L.A.J.D.); omololu@akanji.com (L.A.); D.R.Billson@warwick.ac.uk (D.R.B.); 2 Department of Engineering, University of Perugia, Polo Scientifico Didattico di Terni, Via di Pentima 4, 05100 Terni, Italy; pietro.burrascano@unipg.it; 3 Department of Informatics, Modelling, Electronics and Systems Engineering, University of Calabria, Via Pietro Bucci, 87036 Arcavacata, Rende (CS), Italy; marco.ricci@unical.it

**Keywords:** pulse compression, scattering, attenuation, insulation, ultrasonic testing

## Abstract

Experiments have been performed to demonstrate that ultrasound in the 100–400 kHz frequency range can be used to propagate signals through various types of industrial insulation. This is despite the fact that they are highly attenuating to ultrasonic signals due to scattering and viscoelastic effects. The experiments used a combination of piezocomposite transducers and pulse compression processing. This combination allowed signal-to-noise levels to be enhanced so that signals reflected from the surface of an insulated and cladded steel pipe could be obtained.

## 1. Introduction

Corrosion under insulation (CUI) is a significant industrial problem that has been identified as one of major concern [1]. The basic cause is the leakage of water through the insulation and subsequent contact with the surface of the underlying structure (e.g., a steel pipe), which then corrodes. Water can reach the surface of the underlying metal through two different mechanisms. The first is damage caused to the insulation layers, which creates a path to the metallic material underneath. Alternatively, water could diffuse into the insulation and hence reach the metal surface, initiating the corrosion process. CUI tends to occur at low-lying sections where water can concentrate and/or where there are junctions in structures or pipework. This accelerates corrosion of the outer metal surface, leading to pitting, cracking, and the possibility of subsequent failure.

There is an increasing problem with CUI within ageing infrastructure across numerous existing industrial sites. Sudden failure of pipes or large vessels due to CUI is a serious health and safety issue which, depending on the application, could lead to environmental concerns. There is often a need to strip off the insulation for a manual inspection if CUI is suspected, and additional manpower and costs are associated with this. Corrosion and material failure together are thought to contribute towards 50% of all significant incidents involving hazardous liquid in industrial pipelines [2]. Corrosion processes that occur in CUI include galvanic, alkaline, acidic, and chloride corrosion due to chemicals leaching out from wet insulation [3]. Various techniques exist for the monitoring and inspection of corrosion, and these have been reviewed [4]. They include ultrasound, radiography, eddy currents, and thermography. In many such cases, it is generally assumed that direct access to the metal pipe surface is available, so that the techniques are not designed to be used through insulation layers. One example, the use of ultrasonic guided waves for the detection of pitting due to corrosion, shows promise in the remote detection of corrosion defects [5,6,7], but requires access to a bare pipe and the clamping on of transducer arrays for ultrasonic generation and detection. For insulated pipes, this could be inconvenient and expensive. Radio frequency (RF) signals can also be transmitted along the insulation-filled waveguide formed by a pipe wall and external metallic cladding, and has been used to detect water ingress [8], but suitable access could be a problem in this case also. Low frequency pulsed eddy-current inspection is a through-cladding Nondestructive Evaluation (NDE) technique that has recently been adopted for the NDE of insulted metallic pipes [9,10,11], and has been developed for practical use. The metal magnetic memory (MMM) technique also shows promise for detecting buried pipelines using the earth’s magnetic field, but this is a small effect which would be difficult to use on complicated metallic pipework within an industrial plant [12].

Ultrasound does not seem to have received much interest as an NDE tool for CUI detection through thick sections of insulation. This is probably due to high transmission losses for many common insulation materials, and the wide range of acoustic properties which can vary substantially from one insulation material to the next. This paper thus investigates the possibility that frequencies in the 100–400 kHz range could be used for this task. This would use a combination of transducers with the appropriate bandwidth and matched-filter techniques to improve signal to noise ratios (SNRs). It might then be possible to penetrate significant distances into very attenuating insulation materials. If so, then ultrasound could potentially measure surface topographies caused by pitting of the outer pipe wall, and could also identify the presence of water via a change in propagation velocity within the insulation itself. The present paper investigates whether ultrasound could penetrate through common insulation materials, and recommends how future development of a practical industrial NDE technique for CUI could be achieved.

## 2. Pulse Compression

Pulse compression (PuC) is the chosen method for dealing with the propagation through insulation-covered pipes. The main aim is to output an estimate of the impulse response close to that retrievable by pulsed excitation, but with a much-improved SNR. PuC methods have been used previously in the context of highly attenuating materials [13,14], but experiments in common industrial insulation materials have not previously been reported. This is because of their highly attenuating and scattering nature, and their low acoustic impedances (arising from their naturally low acoustic velocities and densities, making matching to conventional transducers difficult). This necessitated the use of low ultrasonic frequencies and piezocomposite transducers in the present work.

PuC relies on the use of matched filtering techniques, where a coded signal is transmitted into the insulation across a pre-determined bandwidth, and the pulse compression algorithm is then applied to the recorded output. It is essential that the characteristics of this signal are chosen carefully. There are many options available in terms of the signal type, windowing function and processing algorithm. These have been reviewed for use with ultrasound, for example when used for air-coupling [15]. The choice of waveform is associated with several features: the bandwidth required, whether or not the device has a known centre frequency of operation, and the type of measurement to be performed (e.g., whether temporal or spatial information more important [16,17,18]). In the present case, the piezoelectric transducers will operate over a predetermined bandwidth around their frequency of resonance. In addition, the main requirement in the present measurements is to maximize sensitivity, due to the highly-attenuating nature of the insulation materials.

To gain insight into the PuC algorithm, consider a standard ultrasonic test whereby a short time duration yet high voltage amplitude signal feeds the transmitter transducer having a central frequency of ($f_{0}$). If the amplitude of the signal is such that the onset of any non-linear behaviour is avoided, the system can be thus considered linear and time-invariant. Further, if the duration is short enough to excite the whole transducer bandwidth, the output signal represents a good estimate of the overall measurement system impulse response. Theoretically, the impulse response carries all the information about the system under test, in practice, the performance of an ultrasonic inspection will be limited due to the bandwidth (B) of the whole measurement system and the achieved value of signal-to-noise ratio (SNR). It then follows that different aspects must be considered to design an optimal input signal to distinguish between different reflectors/scatterers and to optimise the SNR. Note that PuC is also likely to reduce the effects of structural noise, and to allow better separation of multi-interface echoes if they are present.

In a conventional pulsed ultrasonic system, a good time resolution requires the input signal to have a short time duration ($T_{pulse}$) leading to a broad bandwidth B. This results in a strict constraint for the product between B and $T_{pulse}$ of the signal, such that $T_{pulse}\cdot B≅1$. In addition, the conventional way to improve the SNR is to increase the excitation signal amplitude, but this has limitations and may not be suitable in industrial environments where voltage levels are restricted for safety reasons. An alternative solution is the use of a coded signal input where the excited bandwidth B and the duration T are not constrained by T·B≅1. PuC uses this approach. It is an effective solution for estimating the system impulse response in poor SNR conditions, such as those encountered in high-attenuation insulation. A coded signal x(t) is defined, which distributes its energy over the frequency range of interest and over a relatively long period of time $T_{PuC}$. This is employed as the input signal to the transmitter. The received signal y(t) is the convolution between the system impulse response h(t) and the coded signal, i.e., y(t)=h(t)∗x(t). A matched-filter ψ(t), defined such that x(t)∗ψ(t)≈δ(t), where δ(t) is the Dirac delta function, compresses the received signal y(t) allowing an estimation of the system response $\hat{h}\left(t\right)$ to be retrieved. If the noise affecting the measurement can be modelled as Arbitrary White Gaussian Noise (e(t)), then the PuC process for a single measurement can be described as:

(1) $$\hat{h}\left(t\right)=y\left(t\right)*\psi \left(t\right)=h\left(t\right)*\underset{=\tilde{\delta }\left(t\right)}{\underbrace{s\left(t\right)*\psi \left(t\right)}}+\underset{=\tilde{e}\left(t\right)}{\underbrace{e\left(t\right)*\psi \left(t\right)}}=h\left(t\right)*\tilde{\delta }\left(t\right)+\tilde{e}\left(t\right)\approx h\left(t\right)+\tilde{e}\left(t\right).$$

It follows that if imaging is needed, the PuC algorithm must applied pixelwise for each acquired signal across the scanned area. It can be also noted that the PuC approach can improve SNR with respect to a standard pulsed excitation while assuring the same or even better range resolution. This happens when the energy of the PuC excitation signal is larger than that of a standard pulsed excitation and the same or a larger bandwidth is excited by the coded excitation. As stated above, for a standard pulse of duration $T_{pulse}$, the excited bandwidth is $B\propto \frac{1}{T_{pulse}}$. If the pulse amplitude is constant and equal to $A_{pulse}$, the pulse energy is $E_{pulse}=A_{pulse}T_{pulse}=\frac{A_{pulse}}{B}$. Instead, for the PuC case, the duration $T_{PuC}$ is not constrained by B and can be arbitrary. If the amplitude is constant and equal to $A_{PuC}$, the PuC energy is $E_{PuC}=A_{PuC}T_{PuC}$ from which we can derive the maximum available SNR gain as Equation (2).

(2) $$SNR_{gain}=\frac{SNR_{PuC}}{SNR_{pulse}}=\frac{E_{PuC}}{E_{pulsed-excitation}}=\frac{A_{PuC}}{A_{pulsed}}\cdot T_{PuC}B=k\cdot T_{PuC}B.$$

Thus, the available $SNR_{gain}$ is proportional to the so-called time-bandwidth product of the coded signal [14], and, for a fixed bandwidth *B*, it can be increased by using a longer duration signal ($T_{PuC}$), while the voltage drive amplitude, i.e., the k factor in Equation (2), can be chosen to suit the system requirements [19].

A wide number of signal pairs {x(t),ψ(t)} are available for use in PuC. These include both binary sequences such as maximum length sequences, Golay complementary sequences, Barker codes, and continuous time signals modulated in frequency such as the various types of chirp signals [15]. The choice of signal pairs for a specific test is linked to many factors: the frequency response of the instrumentation (whether it is based on a device with a resonant frequency for example), the need to bias the signal (as in thermography [19]) and the decision on whether the priority is either SNR or time resolution. Conventional binary codes tend to have bandwidths that are a maximum at DC. This is not effective for piezoelectric devices which have a well-defined centre frequency of operation. Bandpass-like binary codes could be defined starting from conventional ones to overcome this problem. This is the case of inverse repeated sequences introduced in air-coupled ultrasound in [15]. The main choice then is between such binary codes and a chirp signal, as will be illustrated below. Although a fair comparison of windowing functions to be applied on the matched filter ψ(t) lies beyond the scope of this work, some discussion has been included in the next section to gain more insight on the sidelobe problem arising from PuC.

## 3. Apparatus

**Figure 1:**
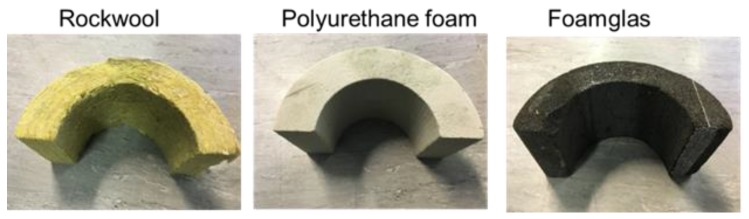
Photographs of the three types of insulation investigated in this work.

There are many types of insulation that are used industrially, but these are classified by ASTM (American Society for Materials Testing, USA) as being available within three basic categories: fibrous, closed-cell expanded foam, and cellular glass types of insulation [20]. Examples of each are shown respectively in Figure 1. Each is in the form to be used on a cylindrical pipe and it has a different type of structure and widely different acoustic properties. Rockwool^TM^ comprises a mineral or rock wool in the form of fibres which are then formed into rolls or slabs of a known density and thickness. It has good thermal and acoustic insulation properties, but is also permeable to water. Moisture is easily retained within its structure—a potential problem for CUI in certain situations. Polyurethane foam is less dense and has better thermal insulation properties than Rockwool, and is more resistant to water ingress due to the closed-pore foam structure. It is structurally stronger than Rockwool. Finally, Foamglas™ is a lightweight, rigid material with gas-filled closed-pore glass cells. It offers a combination of fire resistance, high compressive strength, and impermeability to water.

With reference to the three materials shown in Figure 1, their physical properties vary widely. For example, ultrasonic coupling to Rockwool^TM^ without a liquid intermediate layer introduces losses, and propagation though its fibre/air matrix is complex. Polyurethane is a lossy polymer and the foam contains pores that are gas-filled and scattering. Finally, Foamglas^TM^ has gas pockets surrounded by thin layers of glass which are expected to introduce severe ultrasonic scattering. The use of the PuC technique was designed to deal with these issues.

It is important initially to consider the centre frequency ($f_{0}$) at which to perform ultrasonic measurements on these types of insulation samples, noting that their structures vary considerably. Values of $f_{0}$ below 100 kHz would not be appropriate for the condition monitoring of insulated pipework (e.g., for identifying pitting or water ingress). This is because the corresponding wavelengths (*λ*) would be too long for a reasonable estimation of pitting depth at an insulated metal surface. To give an example, *λ* = 14.8 mm at 100 kHz in water, and while the acoustic velocity in insulation materials can vary (see below), this gives an idea of the wavelengths that could be present in a practical measurement. Conversely, the use of higher frequencies would make ultrasonic transmission more difficult in all three materials because of their composition, and the chosen technique must deal with this fact. The technique would also need to work through the thicknesses of insulation typically used in standard industrial applications. 50 mm has been chosen for study here, but some applications require insulation layers that may be thicker.

**Figure 2:**
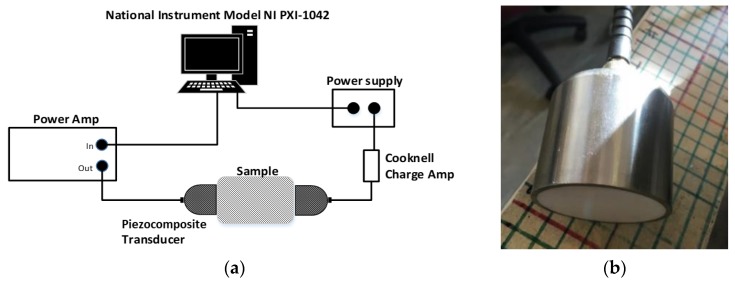
(**a**) Apparatus for low frequency ultrasound at a centre frequency of 150 kHz and a chirp signal swept from 100–200 kHz. (**b**) Piezo-composite transducer.

Initial experiments used dry contact, to avoid the need for couplant that would be absorbed by these materials. These used the apparatus shown schematically in Figure 2a, with the transducers simply pressed against the insulation. Ultrasonic signals were thus transmitted through the samples of insulation shown in Figure 1 in through-transmission. A pair of piezo-composite transducers (see Figure 2b) was chosen for this work, because of their ability to operate over a reasonable bandwidth at these low frequencies. In addition, each transducer was fitted with a quarter-wavelength matching layer for water, which helped transmission into the insulation layers. The transmitter was excited by a chirp signal generated by the National Instruments PXI-1042, incorporating an Arbitrary Waveform Generator NI-PXI 5412 and NI-PXI 5105 digitizer. The output was subsequently amplified via a custom-built power amplifier to 120 V. The piezocomposite transducers were positioned in a through-transmission configuration, and pressed lightly onto either side of a 50 mm thick sample of insulation. The receiver output was connected to a Cooknell CA6C charge amplifier before being input into the same PXI system for processing. Note that later experiments were also conducted in both a water tank and at the surface of a polymer-cladded pipe—the same transducers and instrumentation as that shown in Figure 2a was used for these, with the matching layer well-suited to such situations.

**Figure 3:**
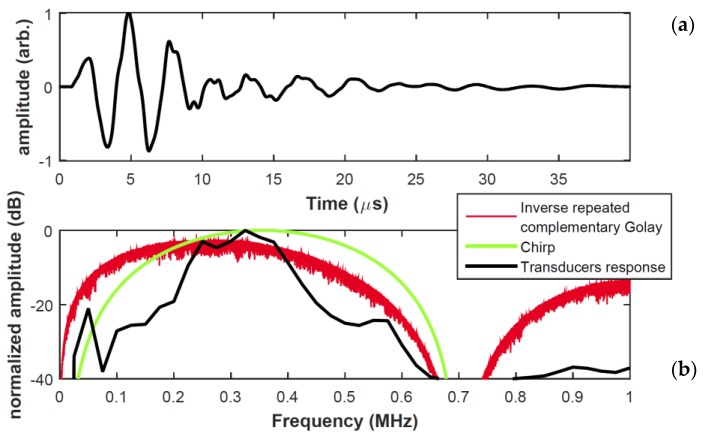
(**a**) The measured impulse response of a 300 kHz piezocomposite transducer. (**b**) Comparison of Inverse repeated complementary Golay and Chirp excitation waveforms for matching to the actual transducer bandwidth.

As stated above, the choice of pulse compression waveform depends in part on the frequency response of the transducers used. To investigate this, the impulse response of the piezo-composite transducers was measured using a transient voltage signal from a Panametrics 5052PRX pulser-receiver, and the results recorded via a Tektronix TDS3032C digital oscilloscope. The impulse response is shown in Figure 3a, whereas Figure 3b depicts the corresponding frequency spectrum (black line). Also shown is the simulated spectrum for two excitation waveforms: an inverse repeated sequence generated from a complementary Golay code and a windowed chirp signal in the 100–700 kHz range. It can be seen that the two types of excitation waveform can be used to produce a response that would excite the piezocomposite transducers efficiently. In the present case, the measurement priorities for a practical NDE tool were for simplicity and improved SNR. However, the use of an inverse repeated complementary Golay code would increase the complexity of a measurement, as two separate signals have to be transmitted at each measurement point. Note also that the complementary Golay code excitation introduces a sideband at frequencies above 750 kHz, which is of lower amplitude in the chirp waveform. For this reason, a set of linear chirp signals with specific values for *B* and *T* were used to optimise PuC for the different piezocomposite transducer pairs used in the measurements to be described below.

To gain insight on the choice on the matched filter ψ(t) employed in the PuC algorithm reported in Equation (1), the mathematical definition of a chirp signal is given in Equation (3).

(3) s(t)=cos(ϕ(t)),

with ϕ(t) being the instantaneous signal phase. Both the properties and the design of a chirp strictly depend on the definition of the instantaneous frequency.

(4) $$f_{ist}\left(t\right)=\frac{1}{2\pi }\frac{d\phi \left(t\right)}{dt}.$$

For a linear chirp signal, the phase is a quadratic function $\phi \left(t\right)=2\pi \left(f_{start}t+\frac{B}{2T}t^{2}\right)\;$, leading to an $f_{ist}\left(t\right)$ being a linear function of time.

(5) $$f_{ist}\left(t\right)=f_{start}+\frac{B}{T}t=f_{0}+\frac{B}{T}\left(t-\frac{T}{2}\right).$$

**Figure 4:**
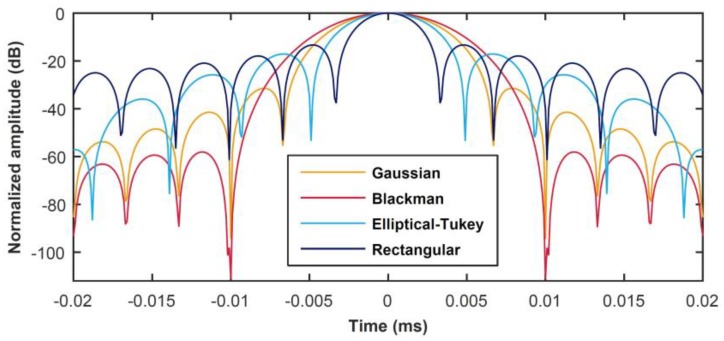
Comparison of the effect of different windowing functions w(t) applied over the matched filter ψ(t) to the PuC output.

Thus, according to Equation (5) a linear chirp signal is a frequency modulated signal whose instantaneous frequency varies linearly within a chosen time duration and bandwidth. Many studies exist in the literature concerning the use of windowing functions w(t) aimed at mitigating the magnitude of sidelobes inevitably arising from the use of coded signals with PuC [21,22,23,24,25,26]. In general, the w(t)’s are applied to the matched filter, i.e., ψ(t)=w(t)·x(−t), whereby x(−t) is the inverted replica of the input signal. Figure 4 shows a comparison of the effect of some well-known windowing functions after applying Equation (1) with y(t)=x(t), i.e., showing the envelope of the auto-correlation function x(t)∗ψ(t)≈δ(t).

With the exception of the rectangular window, the application of the other windowing functions widens the main lobe of the resultant δ(t)’s, although all have a positive smoothing effect on sidelobes reduction. However, the main aim here is to obtain the greatest SNR improvement via PuC rather than distinguish among close scatterers, whereby windowing algorithms are extremely useful. For this reason, a rectangular window having unitary amplitude over the whole duration of the chirp signal has been employed, i.e., w(t) = 1 with t∈[0,T]. Thus, the matched filter is simply the inverted replica of the input signal, i.e., (t) ψ(t)=x(−t), which has been shown to maximise the resulting SNR [21].

## 4. Results

### 4.1. Experiments on Ultrasonic Propagation in Insulation

**Figure 5:**
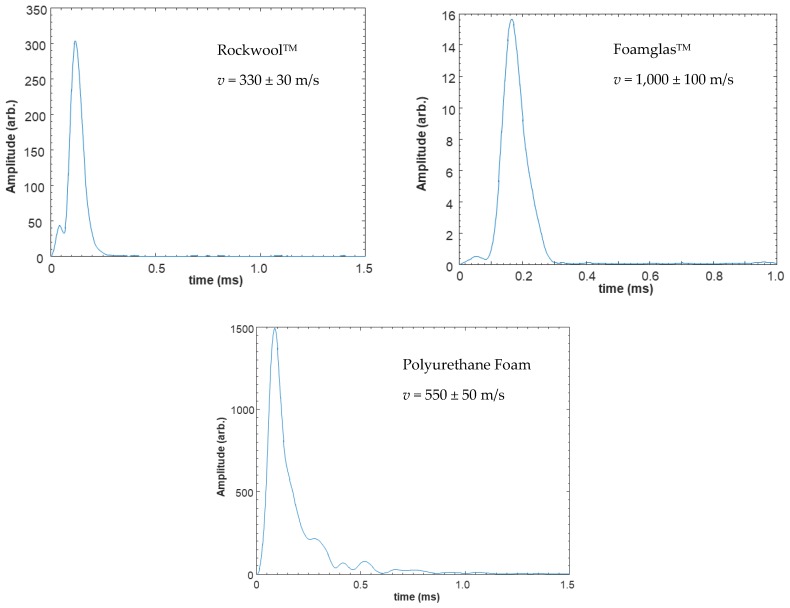
Rectified and smoothed ultrasonic signals transmitted through three samples of 50 mm thick insulation material. The longitudinal velocity (*v*) is shown in each case. The quoted uncertainties arise from the measurement of the sample thickness in each case.

Dry-contact experiments were performed using the apparatus of Figure 2, with a low chirp centre frequency of 175 kHz. The linear chirp excitation signal was swept from 100–250 kHz (*B* = 150 kHz) with *T* = 1 ms. The results from these experiments, in terms of a rectified and smoothed pulse compression outputs, are shown for the three insulation samples in Figure 5. Also shown in each case is an estimate of the longitudinal velocity (*v*) in each material which was derived from these measurements. As might be expected, Rockwool^TM^ had the lowest velocity, polyurethane foam was higher, and Foamglas^TM^ had the highest value at 1000 m/s.

**Figure 6:**
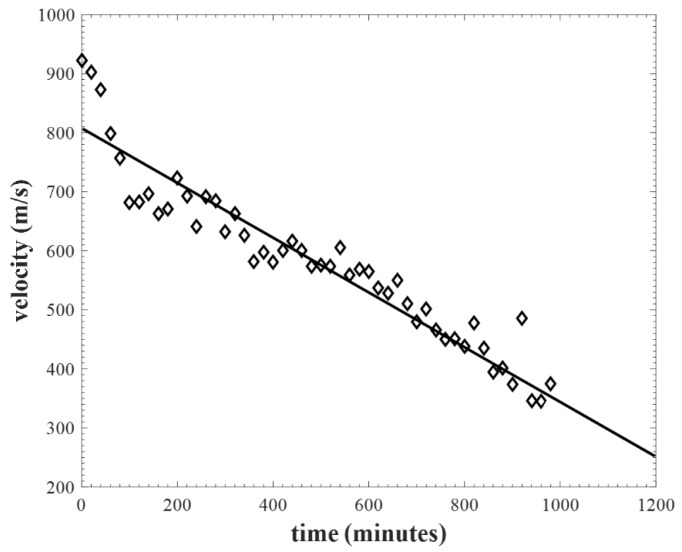
Longitudinal wave velocity decreasing as a Rockwool insulation sample is first saturated with water, and then left to dry out with time at room temperature.

An experiment was also conducted to determine the effects of water when absorbed by Rockwool^TM^ insulation. To this end, a 50 mm thick sample was left in water for 24 h so as to be totally saturated. It was then left to dry naturally at room temperature (20 °C) and the longitudinal velocity (*v*) measured using the apparatus of Figure 2a. The results are shown as a function of time in Figure 6. It can be seen that *v* decreases rapidly initially, but then settles into an approximate linear decrease with time, as indicated by the best fit line shown. While this is only a qualitative result, it does indicate that there is a large change in acoustic velocity with water ingress for Rockwool, and this could thus be used as a good indicator of the presence of water within this insulation material. The structure of the other two materials (polyurethane foam and Foamglas^TM^) is of a closed-pore nature, and hence water retention would not be as marked as that observed for Rockwool^TM^, although this was not measured in this work.

### 4.2. Experiments in Wet Rockwool Insulation

**Figure 7:**
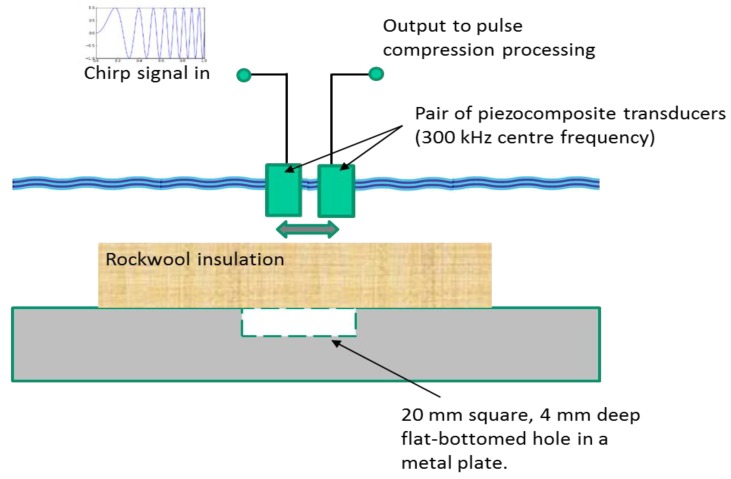
Apparatus used to record pulse compression ultrasonic data from metal surfaces covered in wet insulation layers.

The aim of these second set of experiments was to demonstrate the use of pulse compression ultrasound in the measurement of pitting of a surface when Rockwool insulation was wet—a common situation that would lead to CUI. While the dry contact results above were obtained at *f_0_* = 175 kHz, it was thought that a higher frequency would be needed if the detection of pitting was to be successfully accomplished with a reasonable resolution. The measurements below were thus performed using a pair of 25.4 mm diameter piezo-composite transducers with resonant frequency equal to 300 kHz, again fitted with a matching layer for water. As before, a chirp signal was used to drive the transmitter, this time with a frequency sweep from 200–400 kHz (*B* = 200 kHz and *f_0_* = 300 kHz) and with *T* = 1 ms. Steel samples with surface depressions were fabricated, with known surface contours, and a layer of Rockwool placed over the affected area. The transducer pair, positioned side by side at a centre-to-centre distance of 75 mm, could be scanned in unison horizontally over the sample surface, as shown in Figure 7. Similar instrumentation to that shown earlier in Figure 2a was used to record signals reflected from the metal surface in a pitch–catch arrangement, but now the transducers were immersed within a water tank, and their position controlled using an *x*–*y* stage under control of an external PC. In this way, either single waveforms could be captured at specific locations, or images of sample surface profiles obtained by measuring changes in delay time from transmitter to receiver. The pulse compression output was typically in the form of a smoothed, rectified response from which this travel time could be estimated. Figure 7 indicates an experiment where a sample of 20 mm thick Rockwool was placed over a 4 mm deep, 20 mm wide flat-bottomed square hole, machined into a flat steel surface.

**Figure 8:**
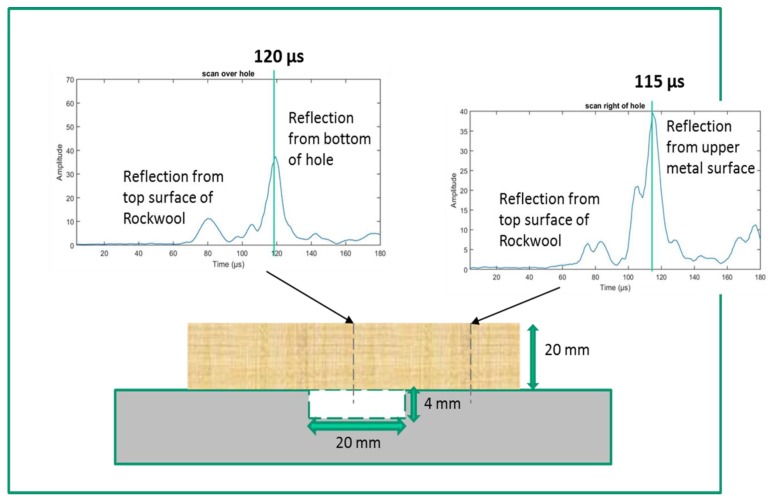
Pulse compression outputs for two locations above a metal surface containing a 4 mm deep flat-bottomed hole. The sample was covered in a 20 mm thick layer of water-saturated Rockwool insulation.

Figure 8 shows the result of scanning the transducer pair over the flat-bottomed hole, where the response at both locations above the top surface and in the presence of the 4 mm deep hole are presented. A pulse compression output waveform is presented at the two locations indicated by the dotted vertical lines. Consider first the one at the left of the figure. When the transducer pair was located over the centre of the 4 mm deep flat-bottomed hole, it can be seen that there were two main peaks in the pulse compression output—an initial peak at 80 µs, which is due to reflection from the top surface of the Rockwool insulation, and a later peak at 120 µs, which is thought to arise from a reflection from the bottom of the hole. To the right of the figure is the pulse compression output when the transducer pair was moved to a position away from the location of the hole. In this case, the reflection from the top of the Rockwool insulation is still visible at 80 µs, but now the subsequent pulse-compression peak has is at 115 µs. The estimated difference of 5 ± 1 µs between the arrival time of the maximum amplitude in each case (120 µs vs. 115 µs) is consistent with a difference in path length of 8 mm (twice the hole depth) between the flat surface and defect regions, where the expected difference in time would be 5.4 µs. The reason for the complicated nature of the arrivals in both waveforms, with double peaks, is thought to arise from inhomogeneity and slight changes in thickness of the Rockwool insulation across the beam diameter, but further complicated by the fact that the width of the transducer beam would be comparable to the width of the hole. Note also that interpretation of the waveform at the hole location is complicated due to the fact that the wavelength at 300 kHz is ~5 mm. Hence, a clear reflection from the underside of the insulation is unlikely to have been resolved in the presence of a signal reflected from the bottom of the hole (which is only 4 mm deep). These measurements also illustrate that transmission though the Rockwool sample could be complicated due to its inhomogeneity, and this fact will need to be taken into consideration in future practical measurements. These experiments do, however, show that transmission through the insulation had been achieved, and that the presence of the hole has been detected.

It is interesting to discuss the minimum depth of hole that could be detected. Pulse compression is a good method for improving depth resolution, by improving SNRs. In our case, the measurements indicated that the depth resolution was ±0.7 mm. It is thus likely that the minimum hole depth detectable would be ~1 mm. This to some extent would depend on the type and thickness of insulation. Thicker or more attenuating insulation layers would not only decrease SNRs but also tend to remove higher frequencies from the signal, affecting bandwidth and hence defect detectability. The longitudinal velocity in the insulation is unlikely to affect this measurement unduly. This will be the subject of future work.

**Figure 9:**
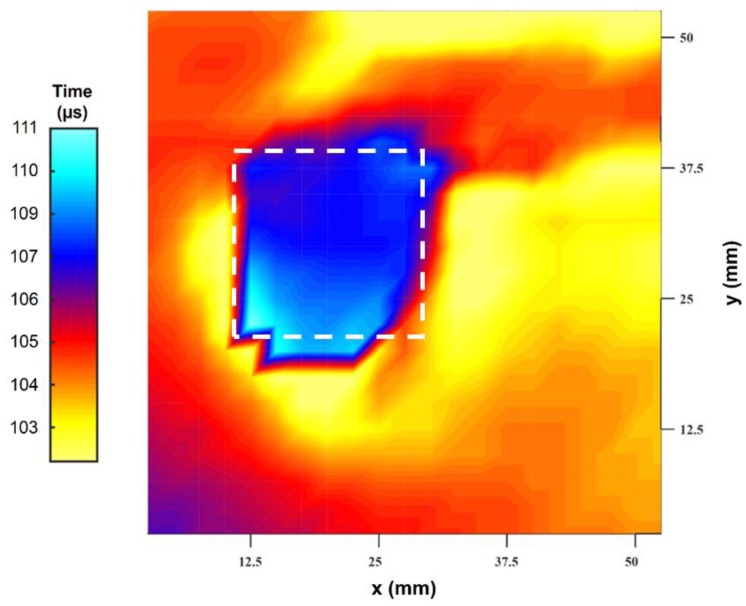
Image of a 20 mm wide hole of 4 mm depth when covered by water-saturated Rockwool insulation. The image was formed using time delay data. The colour scale is arbitrary. The dashed white square outlines the size and location of the artificial flat-bottomed hole.

The piezo-composite transducer pair was now scanned over a horizontal area encompassing the artificial defect intended to simulate surface corrosion. The pulse compression output could be used to provide a depth profile of the hole by plotting the time delay of the second pulse compression peak against location in an *x*–*y* scan. The result is shown in Figure 9, and the presence of the hole is clearly visible. There are some artefacts within this image, and these could be caused by several factors: for example, the transducers are parallel and their beams overlap in a complicated fashion, and both their individual diameters and their separation distance are not negligible in comparison to the width of the defect. However, it is clear that the defect can be detected using this simple arrangement.

### 4.3. Experiments on an Insulated and Cladded Industrial Pipe

**Figure 10:**
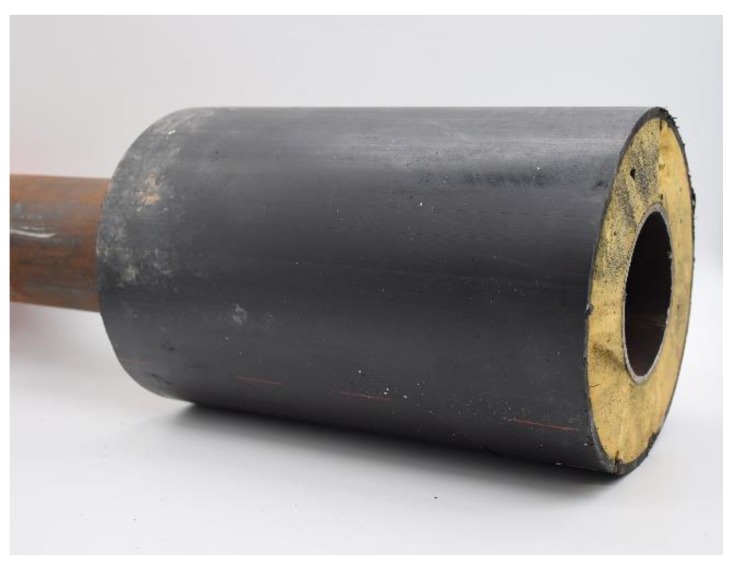
Photograph of the industrial insulated pipe sample.

A final experiment was conducted on a real insulated steel pipe sample. This sample, shown in the photograph of Figure 10, was designed for use in steam distribution applications. It had a 2 mm thick steel pipe of 40 mm radius coated with a 55 mm thick polyurethane foam insulating layer, which in turn was surrounded by a 2.5 mm thick polymer cladding layer. 

**Figure 11:**
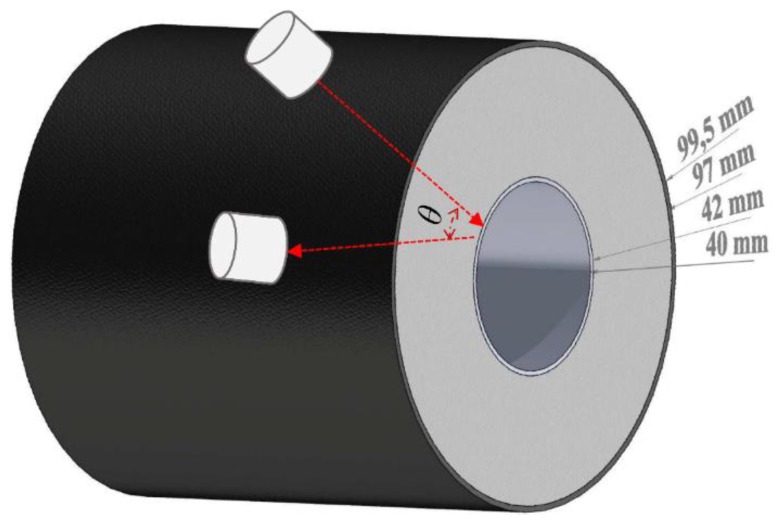
Schematic diagram of the industrial pipe illustrating transducer placement.

A schematic diagram of this pipe, complete with the dimensions of the different layers is shown in Figure 11. The two transducers operating in pitch–catch mode are shown as the white-coloured cylinders, noting that these were the same transducers as used in the water immersion experiments above. They were arranged in a pitch–catch configuration at the surface of the outer thin polymer protective cladding layer, and propagated ultrasonic signals were collected at a range of different transducer separation angles *θ* as shown. Here, *θ* = 25° represents the angle at which the transducers were positioned so that they were touching laterally (i.e., with their geometric centres being 25.4 mm apart). Note that pulse-echo operation with pulse compression is typically difficult because of the long excitation waveform durations, which overloads receiver amplifiers; hence, we chose only to operate in pitch–catch mode. It was also important to provide a set of measurements at various angles, so as to prove that the ultrasonic signal had been reflected from the steel pipe, and not guided around the cladding from source to receiver—this can be calculated by considering time-of-flight and the estimated speed of sound in the insulation. The red dotted arrows indicate the ultrasonic transmission path in pitch–catch mode. Note that a standard couplant for ultrasonic testing was used, and there was no need to fit a curved ‘shoe’ to operate at the cladding surface. Further tests (detailed below) were also performed to confirm that the signal was indeed travelling through the insulation, and not via a guided mode along the circumference of the cladding. This may occur in other samples not tested here, and would need to be taken into account in any practical test [27]. Thickness gauging was not possible in the insulated pipe examined, as the pipe wall thickness (2 mm) was too small compared to the wavelength in steel at 300 kHz (~19 mm). The aim here was to demonstrate penetration through the insulation and reflection from the steel surface.

**Figure 12:**
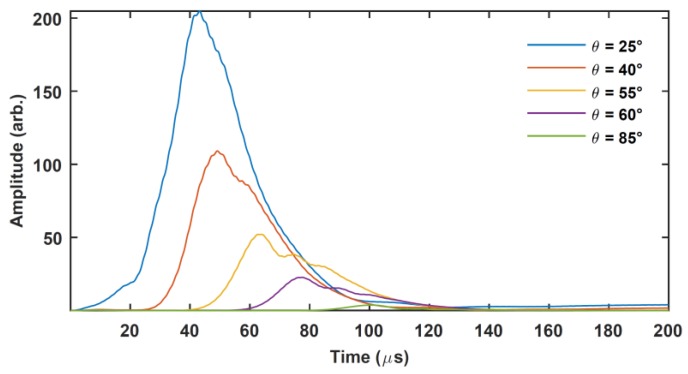
Pulse compression outputs for the sample of Figure 11 at various transducer separation angles *θ*.

Initial tests showed that no recognizable signal could be detected using a conventional nondestructive testing procedure (using the Panametrics pulser-receiver used previously for transducer characterization). However, the results of using the PuC technique on this sample are shown in Figure 12, using a linear chirp with *T* = 1 ms, a chirp centre frequency of *f_0_* = 300 kHz and with *B* = 200 kHz. It can be seen that PuC resulted in the successful detection of signals reflected from the steel pipe outer surface for various values of *θ*. The signal amplitude drops with increasing *θ*, with the expected increased time delay between transmitter and receiver (the two transducer beam axes are not coincident onto the same point on the metal pipe surface but are displaced laterally at increased values of *θ*). Note the excellent SNR of the output at all angles. The measurements in Figure 12 could have included signals arising from a guided-wave mode within the pipe; these have not been identified in this work, but future experiments will identify the types of wave modes present. Note that, at 2 mm, the pipe thickness was too small to allow multiple reflections to occur for thickness gauging for example. Note that the signals could also possibly have been propagating from transmitter to receiver via guided-wave modes within the cladding layer. Other experiments demonstrated that this was not happening—cuts in the insulation to stop such modes had little effect on the received signal, which was primarily due to reflection from the metal pipe. If this situation was to occur, for example if metal cladding was present, then the two propagation modes could be distinguished by the use of a non-linear chirp for PuC [15,18], but this was not necessary here.

The results of Figure 12 demonstrate that the main aim of this work has been achieved: ultrasonic signals can be detected that reflect from a steel pipe surface, in the presence of insulation and cladding. This leads the way towards developing techniques that could detect corrosion of the metal surface, and hence the characterization of CUI. 

## 5. Discussion and Conclusions

It is evident from the above that pulse compression ultrasound can be used to propagate signals though common forms of insulation. Initial dry contact experiments indicated that transmission was possible in various insulation types, provided pulse compression was used in combination with piezocomposite transducers, the latter providing sufficient bandwidth over the 100–400 kHz frequency range studied here. It was also shown that for Rockwool the acoustic velocity changed dramatically with water content, but this has not been measured for other insulation types. This led to a second set of experiments in water immersion, to further refine the technique in pitch–catch mode, and to determine the possibility of imaging of pitting through a waterlogged Rockwool layer. This demonstrated that imaging of a 4 mm deep depression in a metal surface was possible, even when such a feature was covered in a Rockwool layer saturated with water. A further experiment showed that different surface depth features could be measured using time of flight.

The initial experiments indicated that tests on an actual industrially-relevant insulated pipes could be successful, and this has been shown to be the case for a centre frequency of *f_0_* = 300 kHz. The results show that penetration through a 55 mm thick insulation layer was possible with an excellent SNR. Further experiments will continue to refine this technique using pulse compression and scanned transducer locations to produce images, either via manual placement or by using arrays, currently under development. While thickness gauging was not possible in the insulated pipe examined, future work which will involve both thicker steel pipes and metallic cladding.
